# Microstructural and nanoindentation study of TaN incorporated ZrB_2_ and ZrB_2_–SiC ceramics

**DOI:** 10.1038/s41598-022-17797-6

**Published:** 2022-08-12

**Authors:** Seyed Ali Delbari, Abbas Sabahi Namini, Seonyong Lee, Sunghoon Jung, Jinghan Wang, Sea-Hoon Lee, Joo Hwan Cha, Jin Hyuk Cho, Ho Won Jang, Soo Young Kim, Mohammadreza Shokouhimehr

**Affiliations:** 1 Department of Engineering Sciences, Faculty of Advanced Technologies, University of Mohaghegh Ardabili, Ardabil, Iran; 2 Department of Materials Science and Engineering, Research Institute of Advanced Materials, Seoul National University, Seoul, 08826 Republic of Korea; 3 Advanced Nano Surface Department, Korea Institute of Materials Science, Changwon, 51508 Republic of Korea; 4 Division of Powder/Ceramics Research, Korea Institute of Materials Science, Changwon, 51508 Republic of Korea; 5 Innovative Enterprise Cooperation Center, Korea Institute of Science & Technology, Hwarangro 14-gil, Seongbuk-gu, Seoul, Republic of Korea; 6 Department of Materials Science and Engineering, Institute of Green Manufacturing Technology, Korea University, 145, Anam-ro Seongbuk-gu, Seoul, 02841 South Korea

**Keywords:** Structural materials, Ceramics

## Abstract

This study assessed the sinterability and microstructure of ZrB_2_-SiC-TaN and ZrB_2_-TaN ceramics. Spark plasma sintering at 2000 °C and 30 MPa for 5 min produced both ceramics. The relative density of ZrB_2_ ceramic containing TaN was 95.3%; the addition of SiC increased this value to 98.1%. SiC’s contribution to the elimination of ZrB_2_ surface oxides was the primary factor in the advancement of densification. The in situ formation of hexagonal boron nitride at the interface of TaN and ZrB_2_ was confirmed by high-resolution transmission electron microscopy, field emission-electron probe microanalyzer, X-ray diffractometry, and field emission scanning electron microscopy. Moreover, the in situ graphite might be produced as a byproduct of the SiC-SiO_2_ process, hence boosting the reduction of oxide compounds in the ternary system. The SiC compound had the highest hardness (29 ± 3 GPa), while the ZrB_2_/TaN interface exhibited the greatest values of elastic modulus (473 ± 26 GPa) and stiffness (0.76 ± 0.13 mN/nm).

## Introduction

Combining boron or carbon with a transition metal from the fourth or fifth group of the periodic table produces a category of substances known as ultra-high temperature ceramics (UHTCs) with a high melting point (> 3000 °C)^1–4^. Among the UHTCs, ZrB_2_ possesses a number of intriguing properties, including outstanding hardness, a high elastic modulus, and excellent thermal and chemical stability^5–8^. Its particular qualities make it a suitable material for crucibles, armors, thermal shields, leading edges, turbine blades, and other applications^9–12^. Additionally, ZrB_2_'s strong electrical conductivity makes it an appropriate substance for the production of electrical discharge devices and electrodes^13,14^. However, ZrB_2_ exhibits poor sinterability due to its strong covalent bonds and low self-diffusion. In particular, low oxidation resistance at elevated temperatures and low fracture toughness have constrained the use of undoped ZrB_2_ composites^15–17^. A number of studies have attempted to overcome the aforementioned limitations by utilizing advanced sintering techniques for the manufacture of ZrB_2_ composites and/or integrating appropriate sintering additives into ZrB_2_ composites. In terms of the production process, researchers have shown that the use of advanced sintering techniques (such as spark plasma sintering (SPS)) can improve the densification behavior and mechanical properties of ZrB_2_-based ceramics in comparison to the typical powder metallurgy technique^18–20^. During the sintering procedure, the SPS process applies external pressure and sparking phenomenon to the powder particles, considerably reducing the sintering temperature and residence time^21–23^. Regarding secondary phases, the influence of different metallic binders and additives on the qualities of ZrB_2_ composites has been investigated^24^. Nguyen and colleagues evaluated the effect of sintering temperature on the consolidation behavior of ZrB_2_-SiC ceramics^25^. They incorporated 30 vol% SiC to ZrB_2_ matrix; sintering the samples under 10 MPa for 60 min at three different sintering temperatures (2050, 1850, and 1650 °C) using a hot-pressing technique. They demonstrated that fragmentation and particle rearrangement were two significant densification mechanism routes at 1650 °C, but diffusion was perhaps the most important mechanism path at 2050 °C. In addition, plastic deformation was identified as the predominant consolidation process at 1850 °C. Consequently, a nearly fully dense specimen was produced at a sintering temperature of 2050 °C; its relative density value was ~ 8% percent more than the density of the specimen produced at 1650 °C. High-resolution microstructural studies and X-ray diffractometry (XRD) analyses validated the inertness of ZrB_2_-SiC under the applied sintering conditions. Wu et al.^26^ produced ZrB_2_-SiC-BN ceramic via the reactive SPS from an initial composition of B_4_C, Si_3_N_4_, and ZrB_2_. Transmission electron microscopy (TEM) and scanning electron microscopy (SEM) evaluations demonstrated the development of nano- and micro-sized intergranular hexagonal boron nitride (hBN) during the SPS process. Although the impact of hBN on the average grain size of SiC was negligible, the increased amount of hBN could significantly refine the matrix of the final specimens. Nguyen et al.^27^ prepared ZrB_2_-SiC-AlN ceramics using hot-press technique at 10 MPa at 1900 °C for 120 min; the properties of the products was analyzed in terms of sintering behavior and microstructural features. AlN had a significant impact on the densification behavior of the prepared samples, resulting in ceramics that were nearly completely dense. The thermodynamic analysis, XRD results, and microstructural images all supported in situ production of graphite during hot pressing. In addition, the grains were generally fragmented trans-granularly in accordance with the fracture surfaces of the composites, indicating that the constituent particles were strongly bonded. Ahmadi and colleagues^28^ studied the ZrB_2_-SiC composites containing different amounts of Si_3_N_4_. They utilized hot-pressing as the sintering procedure at 1900 °C to achieve fully dense specimens. The reaction between the Si_3_N_4_ and B_2_O_3_ surface oxide led to the formation of hBN. Furthermore, carbon, ZrB_2_, and Si_3_N_4_, which were created during the pyrolysis of the phenolic resin addition, participated in the reaction, resulting in the in situ production of ZrC and hBN components.

The effect of TaN inclusion on the microstructure and sintering behavior of ZrB_2_ and ZrB_2_-SiC composites was studied in this study. Both specimens were sintered at a pressure of 30 MPa and a temperature of 2000 °C for 5 min using the SPS process. The produced ceramics were analyzed utilizing cutting-edge techniques, such as field emission-electron probe microanalyzer (FE-EPMA), field emission SEM (FESEM), high-resolution TEM (HRTEM), XRD, X-ray fluorescence (XRF), and X-ray photoelectron spectroscopy (XPS).

## Experimental

### Materials and preparation method

To produce ZrB_2_-TaN and ZrB_2_-SiC-TaN ceramics, TaN, SiC, and ZrB_2_ powders were admixed according to the compositions presented in Table S1. The particle size of TaN was ~ 5 µm and its purity was 99.5%. In addition, the purity of SiC and ZrB_2_ was 99.2% and 99.8%, respectively, and their particle sizes were ~ 3 µm and ~ 2 µm, respectively. The primary mixtures were dispersed in ethanol and ultrasonically blended for 80 min. The mixtures were completely dried on a magnetic hot plate and in an oven. The mixtures were then loaded into graphite molds and SPSed at 30 MPa and 2000 °C for 5 min. The surfaces of the prepared ceramics were polished using abrasive sheets (numbered 80 to 5000) after the graphite foils were removed. Using diamond grinding disks, the residual surface scratches on the composites were completely removed during the polishing process.

### Analyses and characterizations

The relative density of the SPSed composites was calculated as the proportion of the bulk density to the theoretical density. The crystalline structures of the as-sintered ceramics were verified by XRD (Bruker D8 Advance) analyses. FESEM (Zeiss, SUPRA 55VP) and HRTEM (JEOL JEM-2100F) were utilized for microstructural evaluation, whereas FE-EPMA (JEOL JXA-8530F) was employed to evaluate elemental distribution of the produced composites. In addition, XPS (VG Scientifics, Sigma probe, Al K source) and XRF (Shimadzu, XRF-1800) were utilized to determine the probable elemental bonding and elemental composition of the ceramics. The HSC software (Version 6) was performed to analyze the likely chemical interactions occurring throughout the SPS procedure. The mechanical properties of the existing phases were measured using a nanoindenter (Agilent G200, USA) with a pyramid-shaped tip. Six impressions were made for each phase, and the pertinent information was retrieved from the load–displacement curves. The variables utilized were a loading rate of 40 mN/S for a holding time of 5 s and a maximum load of 400 mN. Each point's hardness was calculated using the Oliver-Pharr method (see the supporting information). Microhardness of the polished ceramics was measured using an Eseway Vickers tester with a diamond indenter under a load of 0.3 kg for 15 s. Using a Vickers hardness instrument, the testing settings for macrohardness were 30 kg load and 15 s.

## Results and discussion

Figure S1a–c depicts the FESEM images and XRD patterns of the as-purchased raw materials utilized in this investigation. According to the XRD patterns of ZrB_2_ and SiC powders, trace amounts of oxides (ZrO_2_ and SiO_2_) are present. Due to the high reactivity of Zr and Si elements with oxygen, it is frequently reported that ceramic and metallic powders contain surface oxides. Although ZrO_2_ oxide has been found as the only crystalline phase in ZrB_2_ powder, B_2_O_3_ cannot be avoided^29^. According to the literature, the evaporation–condensation kinetics during the sintering process of boride-based ceramics can induce B_2_O_3_ to contribute to grain coarsening^30^. In general, the presence of oxide species has a detrimental impact on the densification behavior of metallurgy-produced composites. Moreover, the existence of surface oxides causes excessive grain development, particularly in procedures requiring a long soaking time; they inhibit the formation of strong bonds between adjacent particles^31,32^. These phenomena diminish the mechanical properties of the manufactured composites. If the existence of a secondary phase results in oxide removal during the sintering process, the final product's quality will be greatly enhanced^33,34^. In addition, the SPS approach for removing some low-boiling point oxides (e.g., B_2_O_3_) has been proven effective^35,36^. In accordance with the XRD pattern of the TaN powder (Fig. S1c), a Ta_2_N-related peak was detected in addition to the TaN regular peaks.

**Figure 1:**
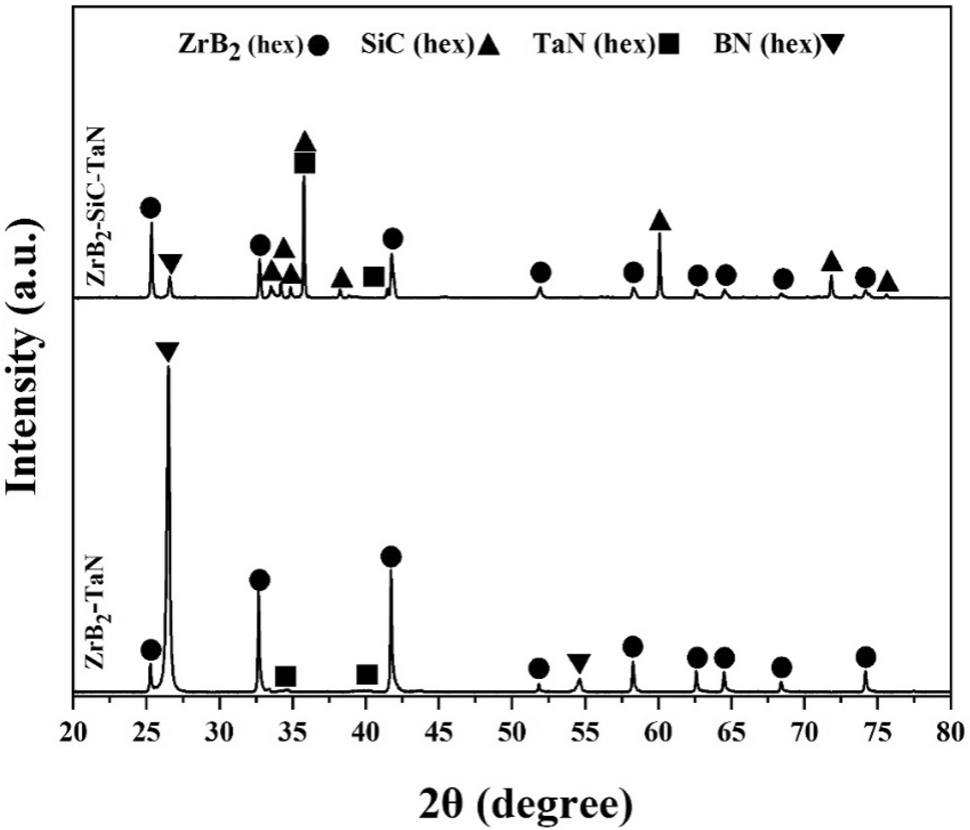
XRD patterns of ZrB_2_-TaN and ZrB_2_-SiC-TaN ceramics.

Initially, the predicted relative densities of ZrB_2_-TaN and ZrB_2_-SiC-TaN ceramics were 95.3% and 98.1%, respectively. The ZrB_2_-TaN ceramic comprised 4.7% residual porosity, while the addition of SiC enhanced its relative density (~ 3%). This improvement is due to the higher sinterability of the ternary system in contrast to its binary equivalent. Figure 1 displays the XRD patterns of the produced ceramics containing and without SiC. The XRD pattern of ZrB_2_-TaN composite reveals two peaks associated with in situ produced hBN at 2θ = 26.5° and 54.6°. In addition to the hBN phase, the original ZrB_2_ and TaN components, no other compound was discernable in the XRD pattern.

A sample can be densified by multiple stages of the SPS procedure. First, a substantial quantity of electrical current flows via the powder particles' contact points, resulting in a local temperature increase. Such phenomena may occur in the ionization/evaporation/melting of surface oxides as well as some of the primary constituents; one of these phase changes is shown in Eq. (1), when the B_2_O_3_ is present in the initial ZrB_2_ particle melts. It should be noted that local temperature can rise as B_2_O_3_ evaporates in the initial stage^37^. In contrast to B_2_O_3_, the other surface oxides of ZrB_2_ (such as ZrO_2_) are stable at extremely high temperatures^38^. Endurance of ZrO_2_ during sintering has the greatest impact on the sinterability of the ZrB_2_-TaN composite. Indeed, there was no reductant in this system to convert this oxide into other compounds. However, the first mechanism of densification in ZrB_2_-based materials is particle fragmentation and rearrangement occurring at temperatures below 1750 °C. The pressure exerted has also a substantial effect on this mechanism. In addition, neck formation takes place at this stage due to the production of electric arcs and plasma between the powder particles. The second densification step in a ZrB_2_ system occurs at temperatures above 1750 °C, where plastic deformation predominates, resulting in the elimination of a significant fraction of accessible pores among the particles. When the temperature of sintering hits 2000 °C, the diffusion densification mechanism can be activated. At this point, there is a substantial potential that hBN might form in situ. Boron and nitrogen atoms may generate an in situ phase during diffusion of elements at the interface of the ZrB_2_ matrix and the TaN additive. Similar behavior was also observed in the TiB_2_-TiN system.

1 $${\text{B}}_{2} {\text{O}}_{3} \left( s \right) = {\text{ B}}_{2} {\text{O}}_{3} \left( l \right)$$

Figure 1 shows the XRD pattern of ZrB_2_-SiC-TaN composite. Similar to the ZrB_2_-SiC composite, the hBN phase was distinguishable in addition to the original phases. The densification mechanisms could be similar to those of the prior composite. The explanation for why the ternary system has a higher relative density than the binary system is addressed below. As previously indicated, the presence of surface oxides might impede consolidation. Based on Eq. (2), the presence of SiC in ZrB_2_ may result in oxide removal. Consequently, ZrO_2_ and B_2_O_3_ can both react with SiC to produce ZrB_2_ and other gaseous compounds.

2 $$2.5{\text{ SiC }} + {\text{ ZrO}}_{2} + {\text{ B}}_{2} {\text{O}}_{3} = {\text{ ZrB}}_{2} \, + \, 2.5{\text{ CO}}\left( g \right) \, + \, 2.5{\text{ SiO}}\left( g \right)$$

The surface oxide of SiO_2_ can interact with SiC phase (Eq. 3), resulting in the formation of in situ graphite and gaseous SiO. No graphite could be observed in the relevant XRD pattern, indicating that the produced carbon engaged in other processes during the SPS procedure.

3 $${\text{SiC }} + {\text{ SiO}}_{2} = \, 2{\text{SiO}}\left( g \right) \, + {\text{ C}}$$

**Figure 2:**
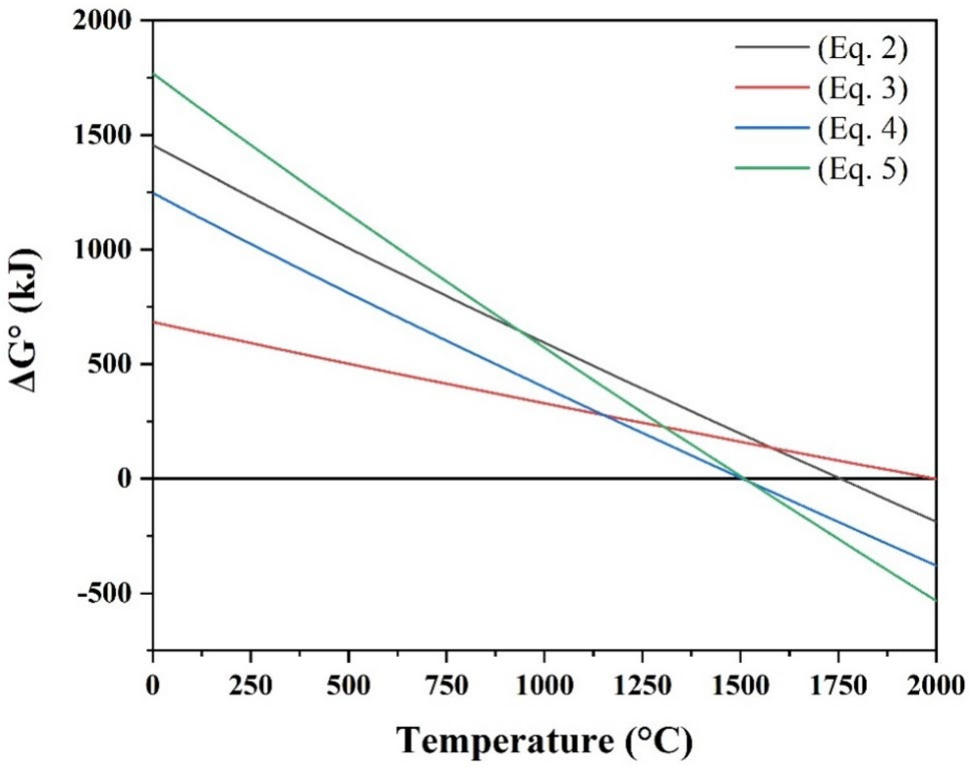
Temperature dependency of ΔG° Eqs. (2–5).

Both Eqs. (4) and (5) imply two plausible consumption reactions for the produced graphite. Carbon can interact chemically with ZrO_2_ and B_2_O_3_ to produce ZrB_2_ (Eq. 4). In contrast, carbon can react with some of the three oxide phases (ZrO_2_, SiO_2_, and B_2_O_3_) to generate ZrB_2_ and SiC in situ. In other words, the presence of SiC in ZrB_2_-TaN composite results in a chain of reactions in which the accessible surface oxides can be reduced to their original phases, hence enhancing the sinterability of the prepared composites. Figure 2 illustrates the dependence of Gibbs free energy (ΔG°) on temperature for Eqs. (2–5). All reactions had negative ΔG° values at 2000 °C, proving their feasibility at the sintering studied conditions.

4 $$5{\text{ C }} + {\text{ B}}_{2} {\text{O}}_{3} \left( l \right) + {\text{ ZrO}}_{2} = {\text{ ZrB}}_{2} + 5{\text{ CO}}\left( g \right)$$

5 $$8{\text{ C }} + {\text{ B}}_{2} {\text{O}}_{3} \left( l \right) \, + {\text{ SiO}}_{2} + {\text{ ZrO}}_{2} = {\text{ ZrB}}_{2} + {\text{ SiC }} + 7{\text{ CO}}\left( g \right)$$

**Figure 3:**
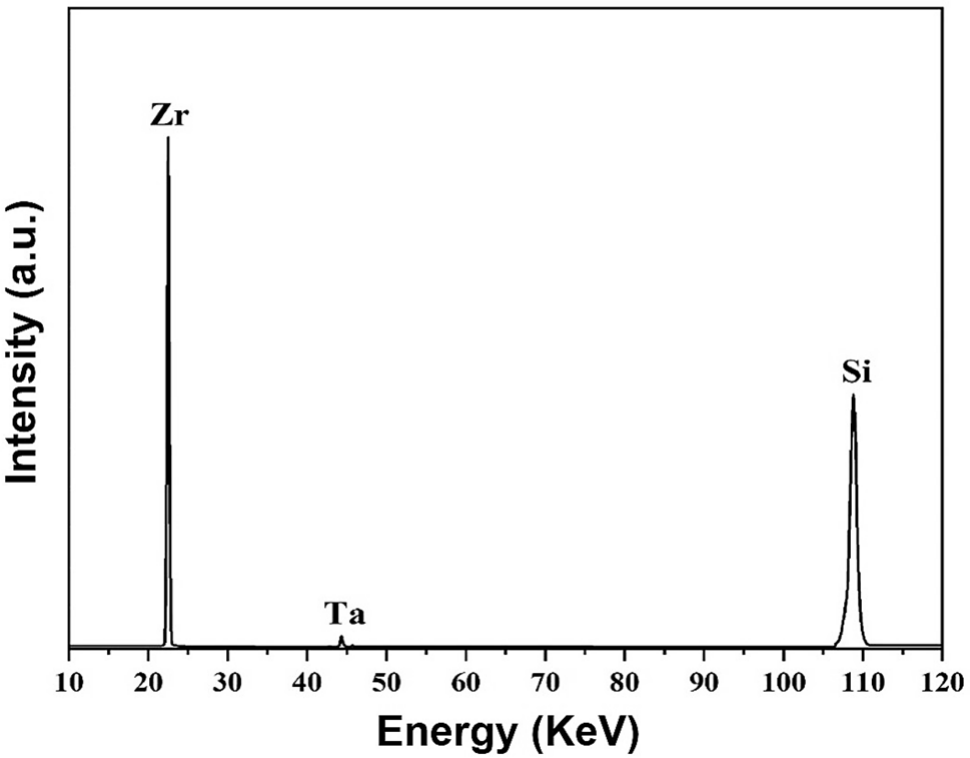
XRF analysis of ZrB_2_-SiC-TaN ceramic.

**Figure 4:**
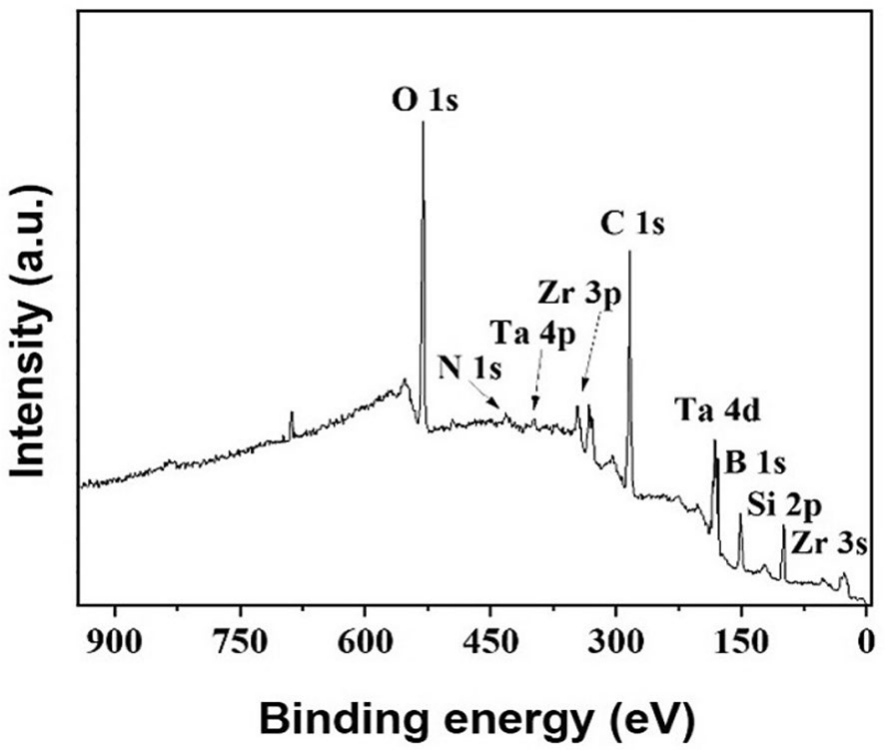
XPS analysis of ZrB_2_-SiC-TaN ceramic.

Using XRF and XPS methods, the ternary structure of ZrB_2_-SiC-TaN ceramic was analyzed. The XRF study (Fig. 3) displays the elemental components of ZrB_2_-SiC-TaN ceramic (Zr, Ta, and Si), excluding those with low atomic mass (B, N, C, and O). Figure 4 exhibits the survey XPS analysis of ZrB_2_-SiC-TaN composite. Figure S2 represents the XPS analysis of C 1s; it reveals two peaks related with the C-Ta and C-Zr binding energies. Interfacial regions between ZrB_2_/SiC and TaN/SiC may create diffusion bonds. However, the XRD analyses were not sensitive enough to detect this effect.

**Figure 5:**
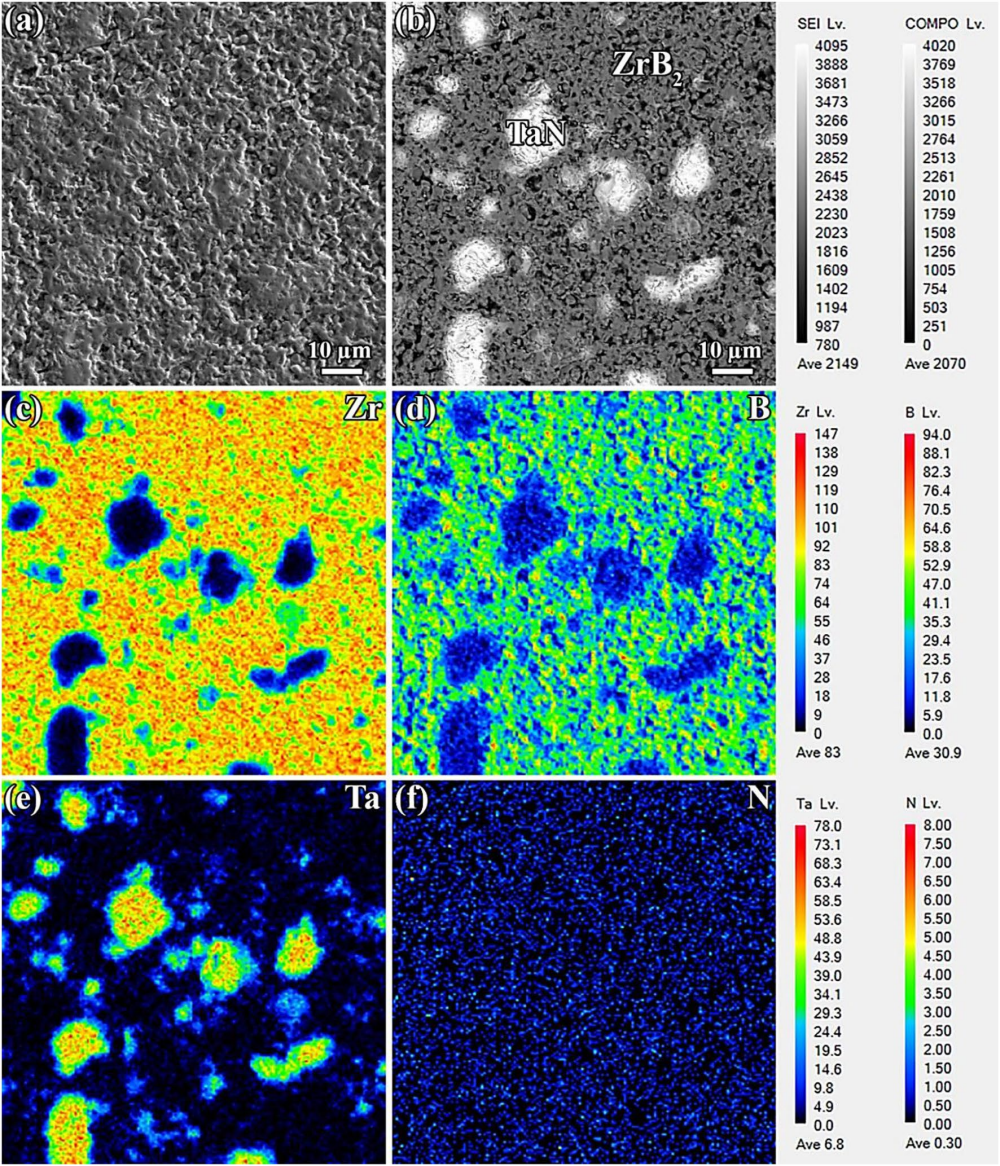
(**a** and **b**) FESEM images of polished surface of ZrB_2_-TaN, and (**c**–**f**) the corresponding EPMA results.

**Figure 6:**
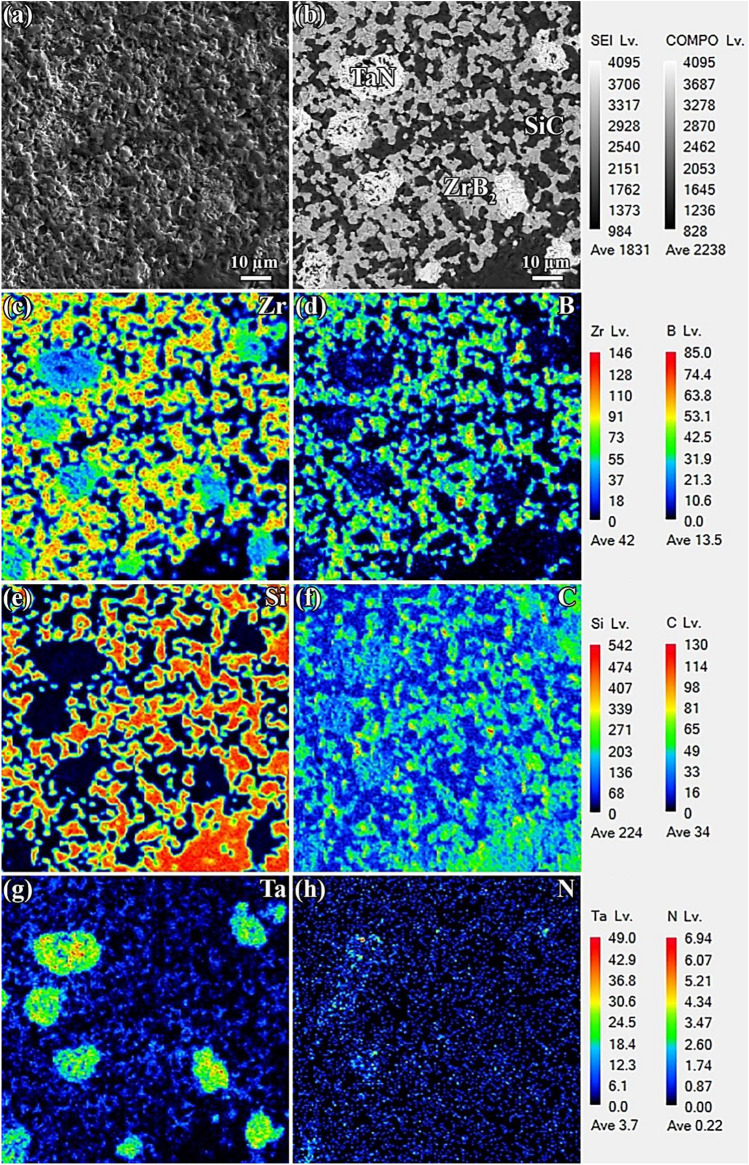
(**a** and **b**) FESEM images of polished surface of ZrB_2_-SiC-TaN, and (**c**–**h**) the corresponding EPMA results.

Figures 5a and 6a demonstrate secondary electron FESEM images of the polished surfaces of ZrB_2_-TaN and ZrB_2_-SiC-TaN ceramics, respectively. It is clear that the ternary ceramic is less porous than its binary counterpart, which is consistent with the relative densities of the prepared ceramics. As previously described, the addition of SiC removes surface oxide species during the SPS process, hence improving the composites' sinterability.

Figure 5a,b demonstrate the FESEM images of the polished surface of ZrB_2_-TaN ceramic. The brightly colored phase is attributable to TaN, which is uniformly distributed within the ZrB_2_ matrix (gray-colored phase). According to the EPMA analysis (Fig. 5c–f), diffusion occurred between ZrB_2_ and TaN compounds at their interfaces. In such areas, the formation of solid solutions (Zr, Ta, and BN) is probable. By comparing Fig. 5d,f, it is possible to deduce that certain regions are rich in both boron and nitrogen. These sites can be seen clearly in Fig. 5d (red zones). This outcome is entirely consistent with the XRD analysis, specifically the in situ formation of the hBN phase as a result of an interaction between ZrB_2_ and TaN constituents at the interfaces. During the elemental diffusion between these two phases, some boron and nitrogen atoms may leave their places in the crystalline structures, contributing to the in situ production of hBN phase.

Figure 6a,b present FESEM micrographs of the polished surface of ZrB_2_-SiC-TaN ceramic. According to the EPMA analysis (Fig. 6c–h), the brilliant, dark, and gray phases are related with the TaN additive, the SiC reinforcement, and the ZrB_2_ matrix, respectively. The uniform distribution of TaN and SiC within the ZrB_2_ matrix indicates that the powder mixture was properly prepared. Similar to ZrB_2_-TaN ceramic, diffusion bonds are visible in the ZrB_2_-TaN and ZrB_2_-SiC interfacial regions. Furthermore, the formation of hBN is possible anywhere boron and nitrogen concentrations are high (Fig. 6d,h).

**Figure 7:**
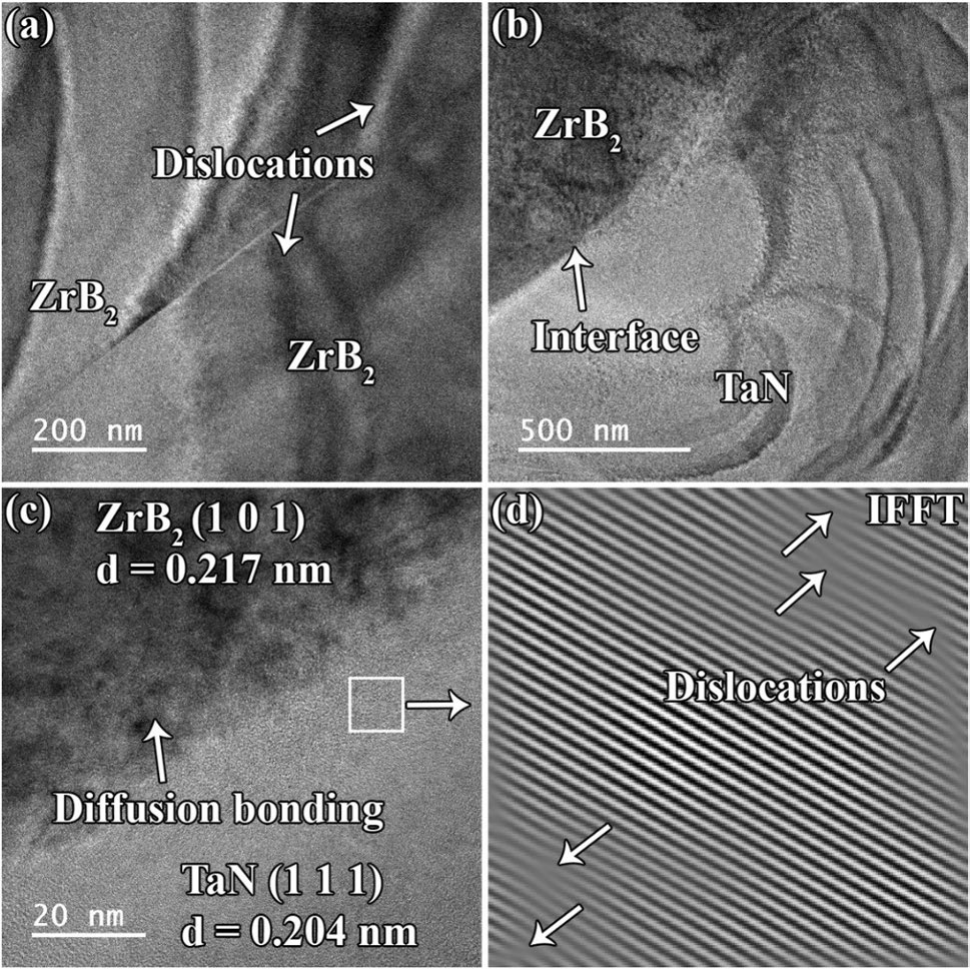
(**a**–**c**) TEM and HRTEM images of ZrB_2_-SiC-TaN ceramics, and (**d**) the relevant IFFT image.

Figure 7a depicts TEM image of the interaction of two adjacent ZrB_2_ grains. It indicates that surface imperfections could be eliminated during the SPS process, allowing neighboring ZrB_2_ particles to form strong bonds. During the sintering process, certain dislocations are formed on both sides of the interfacial region due to the external pressure. The ZrB_2_/TaN interface appears neat (Fig. 7b). However, no visible grain boundary can be detected in the interfacial area image at high magnification (Fig. 7c). Consequently, these two phases may substantially diffuse together, producing a strong connection. The TEM micrograph and inverse fast Fourier transform (IFFT) image reveal the presence of dislocations within the TaN phase (Fig. 7d). In addition to external pressure (as a potential explanation for the production of dislocations), the mismatch between the thermal expansion coefficients of the available ingredients might lead to the formation of dislocations. This behavior would be more prominent during cooling stage of the sintering process.

**Figure 8:**
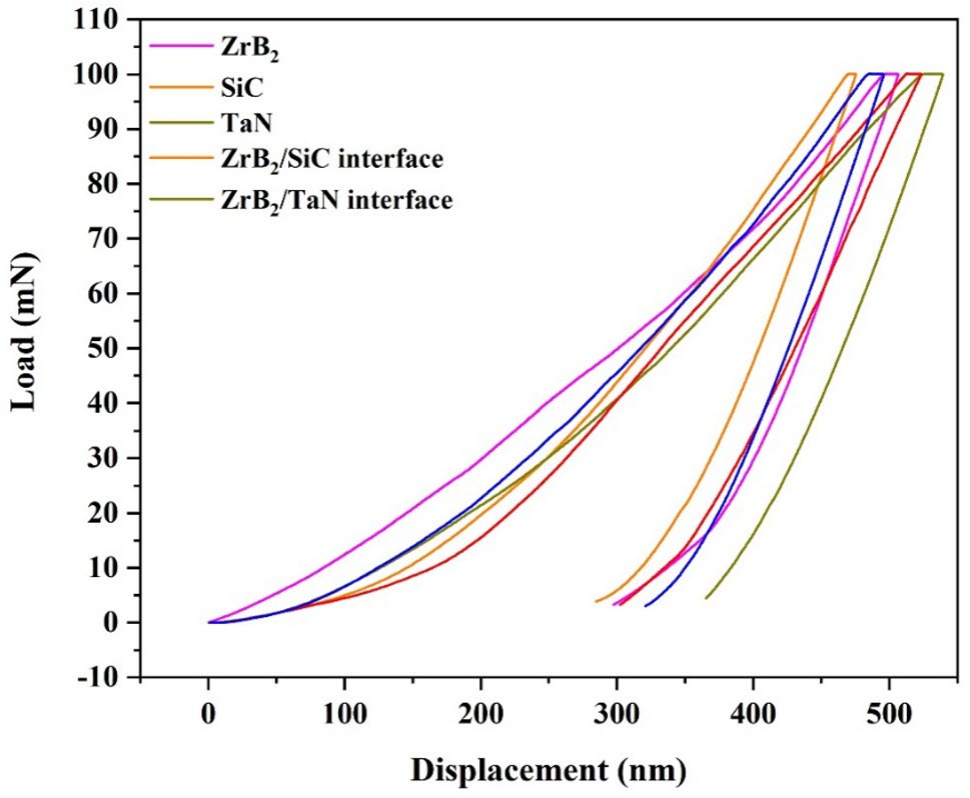
Nanoindentation results of ZrB_2_-SiC-TaN ceramic.

The ternary composite was examined utilizing the nanoindentation technique. Several load–displacement curves relating to different components of this composite are illustrated in Fig. 8. Due to the surface roughness, the calculation of hardness by Oliver-Pharr technique (see supporting information) leads to data propagation^39,40^. Hardness is significantly changed at the areas near the surface when the typical technique is applied to assess the penetration depth. For example, slippage of the indenter's tip may occur under low stresses on protrusions. Consequently, the achieved value for the projected area/indentation depth can be greater than the values obtained on surfaces with depressions or flat surfaces, resulting in lower hardness values. In short, small wrinkling may lead to varied hardness values considering a single phase. According to Table 1, the following is the sequence of hardness, elastic modulus, and stiffness values for ZrB_2_ and interfacial areas: SiC > ZrB_2_/SiC interface > ZrB_2_/TaN interface > ZrB_2_ > TaN (hardness), ZrB_2_/TaN interface > SiC > ZrB_2_ > TaN > ZrB_2_/SiC interface (elastic modulus), and ZrB_2_/TaN interface > SiC > TaN > ZrB_2_ > ZrB_2_/SiC interface (stiffness).

**Table 1:** Elastic modulus, hardness, and stiffness of various phases.

Phases	Elastic modulus (GPa)	Hardness (GPa)	Stiffness (mN/nm)
ZrB_2_	446 ± 16	22 ± 5	0.73 ± 0.11
SiC	471 ± 22	29 ± 3	0.75 ± 0.09
TaN	394 ± 16	19 ± 4	0.74 ± 0.15
ZrB_2_/SiC interface	384 ± 21	25 ± 6	0.56 ± 0.09
ZrB_2_/TaN interface	473 ± 26	24 ± 4	0.76 ± 0.13

Elastic modulus is an intrinsic feature of the materials, which depends on binding forces of atoms and crystalline structure. The formation of strong bonds between adjacent phases enables the attainment of high mechanical characteristics for interface regions. ZrB_2_/SiC, for instance, could attain a hardness value of 25 GPa, which was greater than that of ZrB_2_. It is evident that SiC has a higher hardness compared to ZrB_2_; however, the interface hardness may be significantly lower than surrounding phases if a powerful bonding would not be formed.

The total mechanical work (U_t_) and elastic energy (U_e_) can be measured through calculating the area under each section of load–displacement curves. Afterward, Eq. (6) can be used to estimate the plastic energy (U_p_)^41–43^.

6 $$U_{t} = U_{e} + U_{p}$$

**Figure 9:**
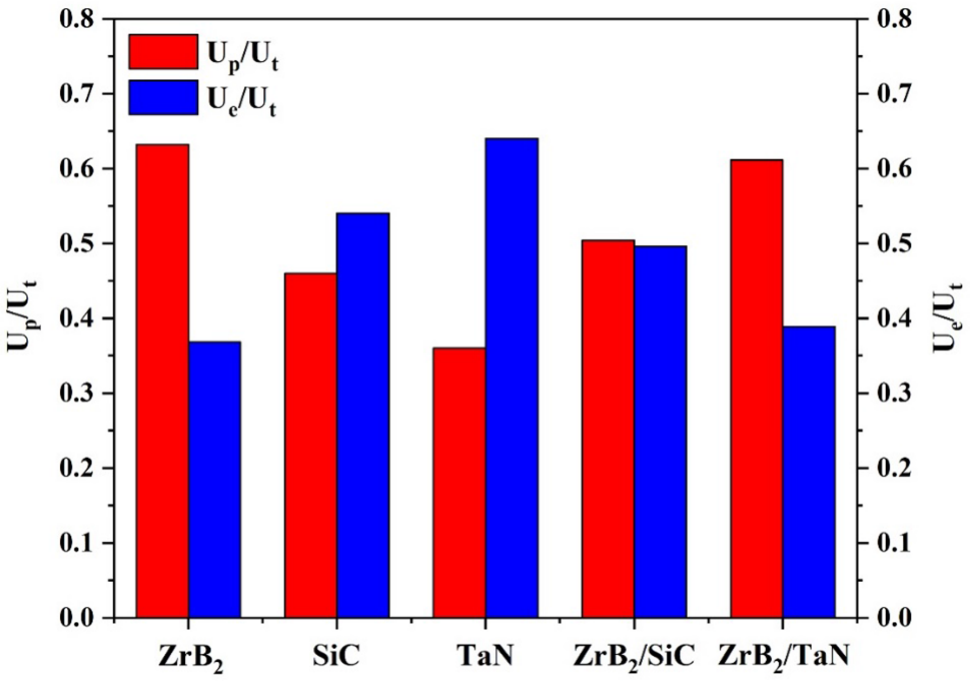
Plasticity index and elastic recovery of different phases in ZrB_2_-SiC-TaN ceramic.

The plasticity index (U_p_/U_t_) and elastic recovery (U_e_/U_t_) of ZrB_2_ and the noted interfaces are calculated. These values (two critical factors for any material) are presented in Table 1 and Fig. 9. Elastic recovery shows the resistivity of a compound against impact loading, while the plasticity index demonstrates the natural response for a component under plastic deformations^41–43^. Based on Fig. 9, ZrB_2_ had the most plastic recovery, while TaN exhibited the highest elastic recovery. Table 2 provides the calculated total energy, plastic energy, elastic energy, plasticity index, and elastic recovery of ZrB_2_-SiC-TaN ceramic phases.

**Table 2:** Calculated total energy, plastic energy, elastic energy, plasticity index, and elastic recovery of different phases in ZrB_2_-SiC-TaN ceramic.

Phase	Total energy U_t_ (mN nm)	Plastic energy U_p_ (mN nm)	Elastic energy Ue (mN nm)	Plasticity index U_p_/U_t_	Elastic recovery U_e_/U_t_
ZrB_2_	21,949	13,869	8080	0.632	0.368
SiC	16,999	7887	9112	0.46	0.54
TaN	21,837	7906	13,931	0.36	0.64
ZrB_2_/SiC interface	19,838	9995	9843	0.504	0.496
ZrB_2_/TaN interface	19,308	11,799	7509	0.611	0.389

Elastic recovery and plasticity index (co-related to nanoindentation elastic and plastic energies) are characterization metrics for ceramic matrix and composites' mechanical performance. The elastic recovery (U_e_/U_tot_) indicates the amount of energy that can be released after loading. U_p_/U_tot_ is related to the inherent flexibility of the material. TaN has the lowest hardness, plasticity index (U_p_/U_tot_ ratio), elastic modulus, and highest elastic recovery as demonstrated in Tables 1 and 2. In addition, despite its intermediate hardness (between SiC and TaN), the ZrB_2_ phase exhibits the elastic recovery of all investigated regions.

Table S2 illustrates the Vickers hardness values of the as-sintered composites. The microhardness of the SiC-free specimen was 15.2 GPa and its macrohardness was 14.3 GPa. However, after the introduction of SiC reinforcement, both of these values increased by ~ 15%. Residual porosity and composition appear to be the two most influential factors in the increases.

## Conclusions

The sintering behavior and microstructural characteristics of ZrB_2_-TaN ceramics with and without SiC reinforcement were investigated. Under sintering conditions of 2000 °C, 30 MPa, and 5 min, spark plasma sintering was applied as the manufacturing procedure. The SiC-free ceramic had a residual porosity of 4.7%, while the SiC-containing ceramic had a residual porosity of 1.9%. Comparing ZrB_2_-SiC-TaN ceramic to a SiC-free specimen, the relative density of ZrB_2_-SiC-TaN ceramic improved most likely due to the influence of SiC on the elimination of surface oxides of ZrB_2_. In addition, it was confirmed that SiO_2_ may be reduced to graphite and gaseous phases by its parent phase (SiC). The generated graphite could also aid in the removal of further oxides during the SPS process. In addition, XRD measurements and microstructural observations showed the in situ synthesis of hBN in both binary and ternary composites as a result of the diffusion phenomenon at the interfaces of ZrB_2_ and TaN. Finally, the SiC phase exhibited the highest hardness value (29 ± 3 GPa), whereas the ZrB_2_/TaN interface exhibited the greatest elastic modulus (473 ± 26 GPa) and stiffness (0.76 ± 0.13 mN/nm).

## Supplementary Information

Supplementary Information.
