# Supplementary material for: Microstructural and nanoindentation study of TaN incorporated ZrB2 and ZrB2–SiC ceramics
Source: Sci Rep. 2022 Aug 12;12:13765. doi: 10.1038/s41598-022-17797-6 (PMC9374782; doi:10.1038/s41598-022-17797-6)
Supplement: Supplementary file 1 — Supplementary Information. [file 41598_2022_17797_MOESM1_ESM.docx]

**Microstructural and nanoindentation study of TaN incorporated ZrB_2_ and ZrB_2_–SiC ceramics**

Seyed Ali Delbari ^a^, Abbas Sabahi Namini ^a,^*, Seonyong Lee ^b^, Sunghoon Jung ^c^, Jinghan Wang ^b^, Sea-Hoon Lee ^d^, Joo Hwan Cha ^e^, Jin Hyuk Cho ^f^, Ho Won Jang ^b^, Soo Young Kim ^f,^*, Mohammadreza Shokouhimehr ^b,^*

^a^ Department of Engineering Sciences, Faculty of Advanced Technologies, University of Mohaghegh Ardabili, Ardabil, Iran.

^b^ Department of Materials Science and Engineering, Research Institute of Advanced Materials, Seoul National University, Seoul, 08826, Republic of Korea.

^c^ Advanced Nano Surface Department, Korea Institute of Materials Science, Changwon, 51508, Republic of Korea.

^d^ Division of Powder/Ceramics Research, Korea Institute of Materials Science, Changwon 51508, Republic of Korea.

^e^ Innovative Enterprise Cooperation Center, Korea Institute of Science & Technology, Hwarangro 14-gil, Seongbuk-gu, Seoul, Republic of Korea.

^f^ Department of Materials Science and Engineering, Institute of Green Manufacturing Technology, Korea University, 145, Anam-ro Seongbuk-gu, Seoul 02841, South Korea.

*** Corresponding authors:**

A. Sabahi Namini ([sabahi@uma.ac.ir](mailto:sabahi@uma.ac.ir)), S. Y. Kim (sooyoungkim@korea.ac.kr), M. Shokouhimehr (mrsh2@snu.ac.kr)

**Oliver-Pharr equation**

$H=\frac{P_{\max}}{A_{c}}$ (1)

$A_{c}=f(h_{c})=24.56\times h_{c}^{2}+0.562\times h_{c}+0.003216$ (2)

$h_{c}=h_{t}-\frac{\varepsilon}{S}\times P_{\max}$ (3)

Where H, A_c_, and P_max_ are hardness, projected area of indentation, and ultimate load, respectively. The contact depth (h_c_) can be metered via Eq. 3. For the used indenter, ɛ is 0.75, S is stiffness, and h_t_ represents displacement of indenter at ultimate load. Besides, Eqs. 4 and 5 present the relations through which elastic modulus is obtained.

$S=\frac{dp}{dh}=\delta\frac{2}{\sqrt{\pi}}E_{m}\sqrt{A_{c}}$ (4)

$\frac{1}{E_{m}}=\frac{1-\nu_{s}^{2}}{E_{S}}+\frac{1-\nu_{i}^{2}}{E_{i}}$ (5)

Where E­_i_, E_s_, ʋ_i_ and ʋ_s_ are elastic modulus of indenter, elastic modulus of sample, Poisson's coefficient of indenter, and Poisson's coefficient of sample, respectively. Finally, δ is a constant value (1.034), which depends on the geometry of the indenter.

**Table S1.** The composition of the prepared composites.

| Composite | TaN (wt%) |  | SiC (wt%) | ZrB_2_ (wt%) |  |
| --- | --- | --- | --- | --- | --- |
| ZrB_2_-TaN | 15 |  | - | balanced |  |
| ZrB_2_-SiC-TaN | 15 |  | 20 | balanced |  |


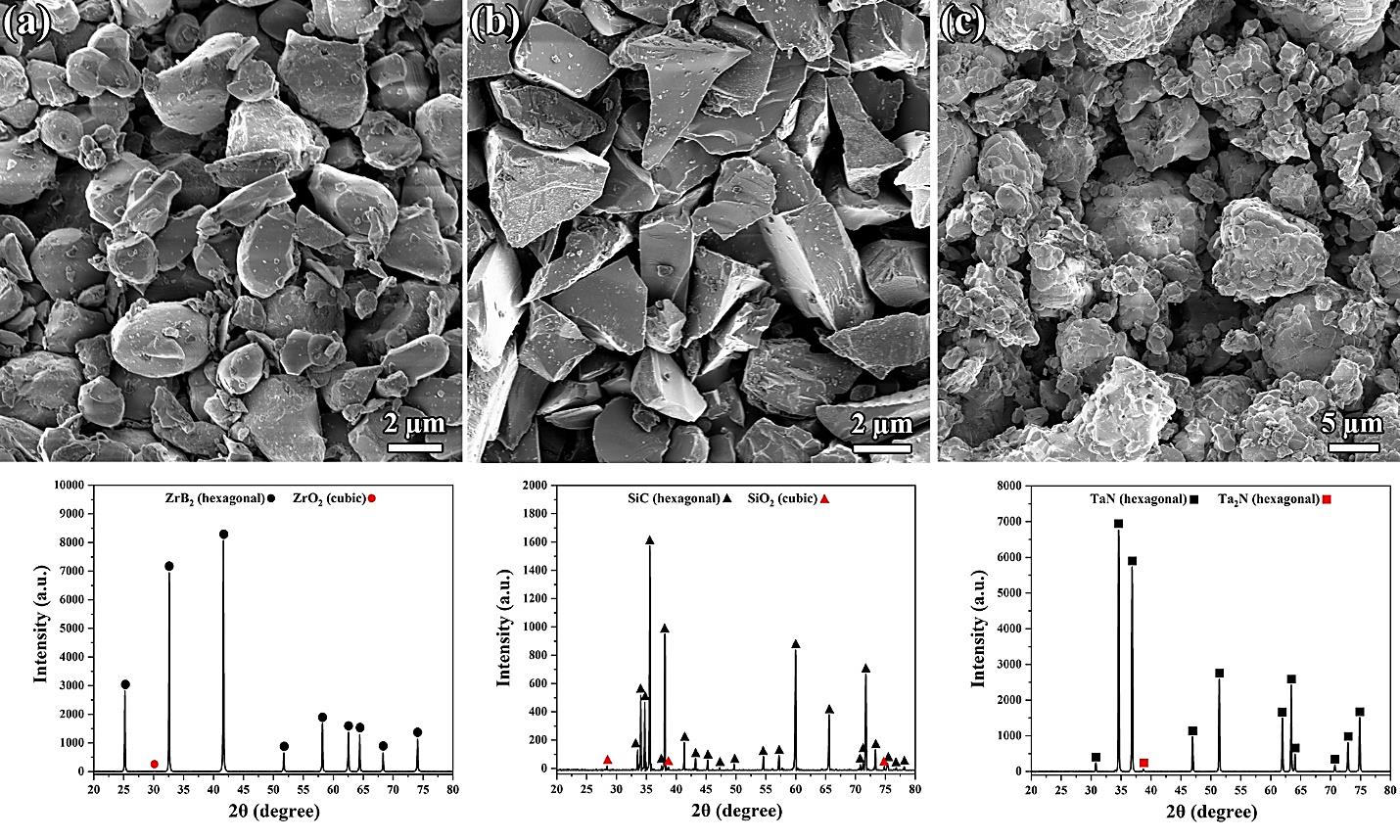


**Figure S1.** FESEM micrographs and corresponding XRD patterns of the starting substances; (a) ZrB_2_, (b) SiC, and (c) TaN. Reprinted with permission from Ref. [S1] Elsevier 2021.

**Table S2.** Micro and macro-hardness values of the prepared composites.

| Composite | Microhardness (GPa) | Macrohardness (GPa) |
| --- | --- | --- |
| ZrB_2_-TaN | 15.2 ± 0.9 | 14.3 ± 0.6 |
| ZrB_2_-SiC-TaN | 17.6 ± 1.1 | 16.4 ± 0.7 |

**
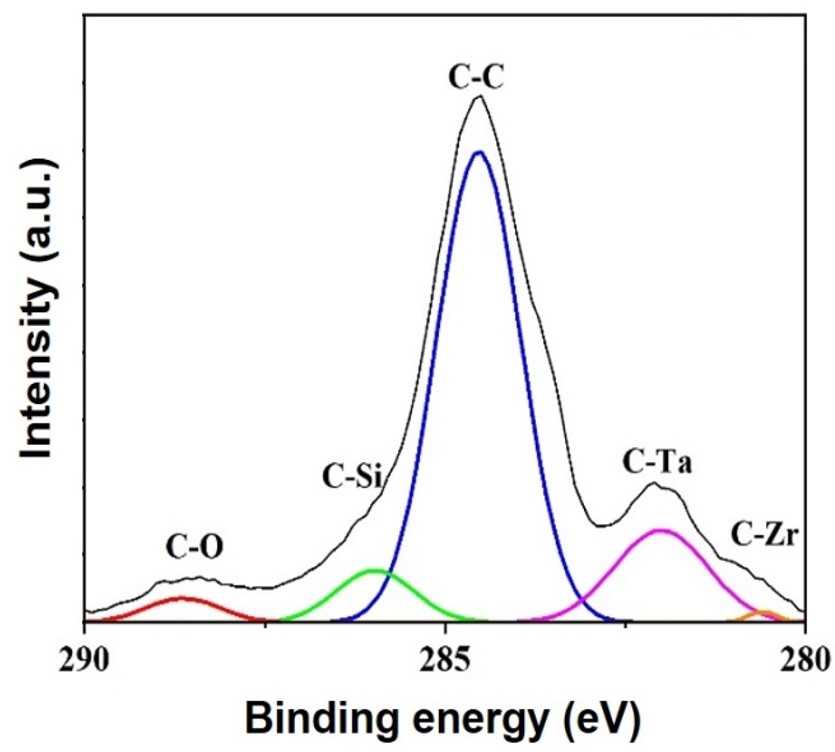
**

**Figure S2.** XPS analysis of C 1s carbon in the ZrB_2_-SiC-TaN ceramic

**Reference**

[S1] A. Sabahi Namini, S. A. Delbari, M. Shahedi Asl, Q. V. Le, M. Shokouhimehr, J. Taiwan Inst. Chem. Eng. 119 (2021) 187-195. Characterization of reactive spark plasma sintered (Zr,Ti)B2–ZrC–SiC composites. https://doi.org/10.1016/j.jtice.2021.02.020.
